# Texture analysis to differentiate anterior cruciate ligament in patients after surgery with platelet-rich plasma

**DOI:** 10.1186/s13018-021-02437-y

**Published:** 2021-04-28

**Authors:** Allan Felipe Fattori Alves, José Ricardo de Arruda Miranda, Sérgio Augusto Santana de Souza, Ricardo Violante Pereira, Paulo Roberto de Almeida Silvares, Seizo Yamashita, Elenice Deffune, Diana Rodrigues de Pina

**Affiliations:** 1 Medical School, Sao Paulo State University Julio de Mesquita Filho, Av. Prof. Mário Rubens Guimarães Montenegro, s/n - UNESP - Campus de Botucatu, Botucatu, SP CEP 18618687 Brazil; 2 Institute of Bioscience, Sao Paulo State University Julio de Mesquita Filho, R. Prof. Dr. Antônio Celso Wagner Zanin, 250 - Distrito de Rubião Junior, Botucatu, SP CEP 18618687 Brazil

**Keywords:** Knee joint, Classification, Magnetic resonance imaging, Texture analysis, Machine learning, Platelet-rich plasma

## Abstract

**Background:**

Platelet-rich plasma (PRP) has been used to favor anterior cruciate ligament (ACL) healing after reconstruction surgeries. However, clinical data are still inconclusive and subjective about PRP. Thus, we propose a quantitative method to demonstrate that PRP produced morphological structure changes.

**Methods:**

Thirty-four patients undergoing ACL reconstruction surgery were evaluated and divided into control group (sixteen patients) without PRP application and experiment group (eighteen patients) with intraoperative application of PRP. Magnetic resonance imaging (MRI) scans were performed 3 months after surgery. We used Matlab® and machine learning (ML) in Orange Canvas® to texture analysis (TA) features extraction. Experienced radiologists delimited the regions of interest (RoIs) in the T2-weighted images. Sixty-two texture parameters were extracted, including gray-level co-occurrence matrix and gray level run length. We used the algorithms logistic regression (LR), naive Bayes (NB), and stochastic gradient descent (SGD).

**Results:**

The accuracy of the classification with NB, LR, and SGD was 83.3%, 75%, 75%, respectively. For the area under the curve, NB, LR, and SGD presented values of 91.7%, 94.4%, 75%, respectively. In clinical evaluations, the groups show similar responses in terms of improvement in pain and increase in the IKDC index (International Knee Documentation Committee) and Lysholm score indices differing only in the assessment of flexion, which presents a significant difference for the group treated with PRP.

**Conclusions:**

Here, we demonstrated quantitatively that patients who received PRP presented texture changes when compared to the control group. Thus, our findings suggest that PRP interferes with morphological parameters of the ACL.

**Trial registration:**

Protocol no. CAAE 56164316.6.0000.5411.

## Introduction

The technological advance of regenerative medicine capacitated the integration of tissue engineering with orthopedics daily practice, allowing prosperous results in the treatment of lesions and diseases [1, 2]. The anterior cruciate ligament (ACL) reconstruction surgery, frequently performed in sportive medicine, is an example of such integration [1, 3]. ACL is important since it maintains the movement and normal stability of knee articulation. ACL is usually injured due to high tension forces of traction and knee torsion. Over 400,000 reconstruction surgeries are performed annually worldwide [4]. Treatments choices consider factors such as patients’ age, knee instability, and the type and sports practiced. Rehabilitation is often recommended but most patients have incomplete recovery which can lead to an inability to return to activities before injury. New strategies have been studied to improve ACL repair and decrease recovering time [4, 5].

Some applications with biological agents have been used to increase treatment effectiveness. The platelet-rich plasma (PRP) is one of the main biological agents investigated for this purpose [6]. PRP is mostly used due to its effects on the stimulation and repair of musculoskeletal injuries [7]. PRP is derived from autologous blood samples and contains suprafisiological concentrations of platelets, growth factors, and bioactive molecules. Those components can promote tissue healing and regulate joint homeostasis through several processes. They promote anabolic cell stimulation, increase in the deposition of the extracellular matrix, reduction in the pro-apoptotic and anti-inflammatory joint environment. It can be easily obtained, prepared, and applied during the ACL reconstruction surgery [6]. PRP can decrease tissue regeneration time which can be very beneficial especially for elite professional athletes [8].

There are several papers that describe the PRP role in the ACL repair after surgery [7, 9, 10]. However, there is divergence in the literature to better understand its mechanism, standardize all variables, and demonstrate quantitatively that PRP produced tissue changes. In this context, the evolution of quantitative methods applied to medical images in which computational tools are used may help to obtain and analyze larger volumes of data to be applied clinically. These techniques allow standardized and trustful measurements that surpass the visual subjectivity interpretation [11]. Spatial resolution and contrast dynamic range have increased in imaging modalities with computed tomography (CT) and magnetic resonance image (MRI). Through those modalities, more information can be extracted from the pixels that composed the regions of interest (RoI). The characterizations made by quantitative analyses have the potential to complement the coming information of molecular biomarkers with the advantage of being non-invasive [12]. Image processing in medicine has been already used to quantitative analyze joints and musculoskeletal tissues [11, 13]. In addition, computational approach such as machine learning (ML) has been used in several fields of medicine such as patient outcome prediction or lesion classification [14–17]. ML is commonly applied to medical images through the analysis of quantitative radiomics features such as those extracted by texture analyses (TA) [18].

There are still contradictions among studies that assess the potential effects of PRP on the ACL. Moreover, there is a lack of quantitative assessments of the intrinsic characteristics of radiological images which demands new approaches in this field. In this work, we propose a new method to quantitatively differentiate ACL of patients that undergone reconstruction surgery with and without the use of PRP. It is important to present new approaches for assessing tissue structure through texture associated with machine learning applied to retrospective MRI exams, which can contribute to assess the success of the procedure (treatment).

## Materials and methods

This retrospective study has been approved by the Institutional Review Board (IRB) of the authors’ affiliated institutions. Proper informed consent was obtained from all patients. The patients included in this study were determined with the following inclusion criteria: patients over 18 years old; with confirmed ACL acute and chronic injury; submitted to ACL reconstruction surgery. The following exclusion criteria were adopted: patients under 18 years old; with any autoimmune disease that causes joint impairment; with positive sorology for hepatitis B or C, HIV, HTLVI/II or Chagas disease. After this stage, patients were conducted to the plasma autologous collection necessary to produce the platelet-derived hormones.

In our study, we utilized different scores to demonstrate knee conditions before and after (3 months) the surgery for both our groups [19–23]. Those scores are as follows: knee circumference, pain, flexion, IKDC index (International Knee Documentation Committee) [24], and Lysholm score [25].

The patients passed through clinical and laboratory triage to test blood transmissible diseases to determine their eligibility according to the autologous donation legislation no. 158 of February 4, 2016 [26]. Approved candidates for clinical screening were referred for autologous whole blood donation, with subsequent segregation of blood components: red blood cells (RBC), frozen fresh plasma (FFC), and platelet concentrate (PC) [27, 28].

The statistical analysis was performed using the PRISM 7 software (GraphPad software, Inc., 2016). Significant differences in the mean values of scores described above were determined using the paired and not paired Student *T* test. We considered them statistically different when the *p* value was < 0.05.

### PRP production and storage

Following the donation, the blood components were processed according to institution standards. The RBC unit was stored in an appropriate cooler. The FFP was frozen in an automated temperature decay system in Thermogenesis® equipment and then transferred to a freezer at −80 ° C. The PC was in continuous agitation until the preparation of the Platelet Derived Hormones (PDH) [27].

The FFC and PC units of each patient were sent to the Cell Engineering laboratory of our institution for the production of PDH. After processing, the cryoprecipitate and the PDH were ready for application [27].

### Study group

After the selection, 34 patients were included in this study. The control group was composed of 16 patients, 9 male and 7 female, mean age of 32 ± 7 and mean BMI (kg/m^2^) of 24.1 ± 6.68. The patients were submitted to the operative procedure of ligament reconstruction with an arthroscopic technique using the gracilis and semitendinosus tendons. Hamstring autograft fixation was performed with metallic or bio-absorbable cross-pin femoral fixation in the distal femur, in the proximal tibia; it was used as delta-type bio-absorbable screw with a metallic staple [29]. In the control group, there was no application of PRP.

The experimental group was composed of 18 patients, 10 male and 8 female, mean age of 29 ± 8 and mean BMI (kg/m^2^) of 29.01 ± 2.87. The group was composed of patients with surgical indication who underwent the same procedure performed for the control group. The difference was that prior to the closure of the operative field, the patient received the application of 4 ml of PRP in loco [28]. All the patients included in this study performed magnetic resonance imaging of the knee 3 months after surgery.

MRI was performed using a 3T scanner (Magnetom Verio, Siemens, Erlangen, Germany) with an 8-channel-knee coil. Turbo-spin-echo (TSE) DP FAT SAT sequences (FOV: 170 × 170 mm; matrix: 384 × 384; slice thickness 2.5 mm; flip angle: 150°; GAP 1; TR/TE = 3550/44; NEX: 2) were acquired on the sagittal view. Total sequence acquisition time was approximately 4 min. In this study, we utilized MRI T2-weighted images for the subsequent steps described below.

### Texture analyses (TA)

#### TA features extraction

Textures are attributes present on images that correspond to a visual pattern or arrangement of structure, usually related to the distribution of pixels. Texture usually contains very significant information about image content which makes it widely used in image processing [30]. Texture analysis (TA) refers to the characterization of regions based on their texture content. TA applied in medical imaging provides a tool to classify images, or to differentiate between healthy and pathological tissues [31].

**Fig. 1:**
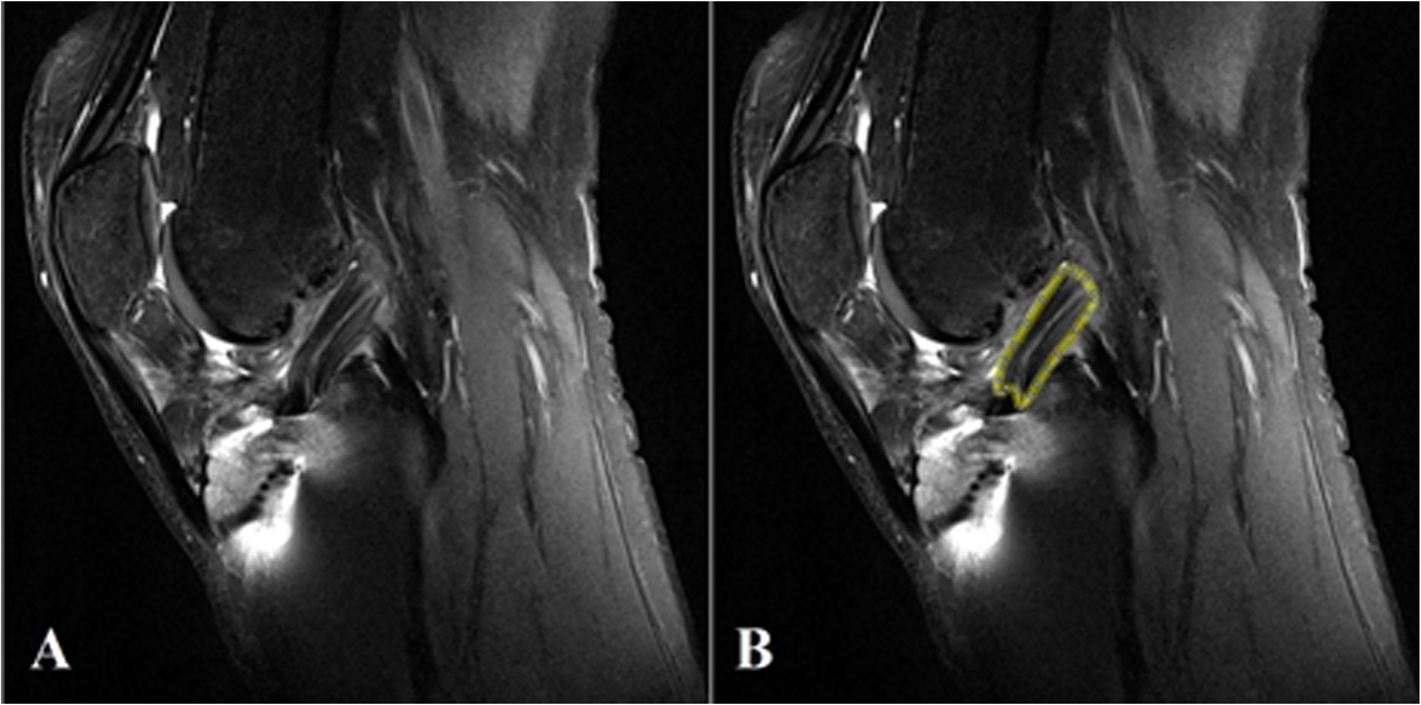
Example of RoI (**r**egion of **i**nterest) selection process. **a**: Original image; **b** image with an ACL (**a**nterior **c**ruciate **l**igament) RoI positioning in Matlab® environment

The TA features extraction was performed in the Matlab® software R2017a. First, we selected the RoIs in sagittal slices of MRI T2-weighted images. The slice with the largest ACL longitudinal section visible in the image was selected (Fig. 1a). The RoIs delimited the region of the ACL of each patient (Fig. 1b). RoI positioning was performed by two experienced radiologists. From the delimited RoIs, 62 texture parameters were extracted, including gray-level co-occurrence matrix (GLCM) and gray-level run-length (GLRL) [32, 33]. GLCM contains the second-order statistical information of neighborhood pixels of an image. GLRL provides a dimensional matrix that calculates the number of adjacent pixels that have the same gray intensity in a particular direction [32, 33].

#### Machine learning analyses and algorithms

After the previous, we transferred the dataset to the Orange Canvas (v 3.18) software. Orange contains a powerful library that includes a selection of machine learning methods, processing methods, and sampling techniques. We used Orange Canvas to perform the analyses in all our extracted texture features dataset [34]. First, the dataset was divided into two independent parts: a training set (75% of the total sample) and a test set (the remaining 25%), with a tenfold cross-validation. Different machine learning classifiers were utilized with our training set, to determine the best combination of features to achieve the highest accuracy. We used the following learning classifiers:

Logistic Regression (LR) which is used to analyze the relationship between continuous or categorical predictive variables (explanatory or independent), and a categorical outcome that produces binary variables responses (dependent). LR estimates the probability of the dependent variable to assume a certain value as a function of known variables [35].

Stochastic gradient descent (SGD) which is a standard algorithm to optimize complex functions iteratively. SGD has a high impact on machine learning with its optimization method for unconstrained problems. It approximates the true gradient by considering a single training example. The algorithm works iteratively over the training examples updating the model parameters with each iteration [36].

Naive Bayes (NB) which is based on the Bayes’ theorem and the maximum posterior hypothesis, assuming that the effect of an attribute on a given class is independent of the values or other attributes called “conditional independence.” The classification does not require an accurate probability estimate as long as the maximum probability is assigned to the correct class [18].

Gain ratio and Gini index were used to rank all features according to their correlation with each class (Raileanu and Stoffel, 2004; Stoffel and Raileanu, 2001). Thus, from the 62 texture features, we selected the five features that achieved the highest scores for classification within each machine learning classifier. To determine how efficiently the models classified our groups, we utilized quantitative parameters such as the area under the receiver operating characteristic curve (ROC), accuracy (CA), F-score (F1), precision, and sensitivity.

## Results

### Texture and machine learning analyses

RoIs were positioned on the sagittal plane along the largest diameter of the ACL by two experienced radiologists. The localization agreement between them was excellent, with less than 2% of area variation in the selection of the ACL in the MRI slice. From each RoI localization, 62 texture features were extracted. Those features were processed by each classifier, and then the five best-ranked features were selected. After establishing a model through the training set, the classifiers were applied on the independent test set, with the following quantities: area under the ROC curve (AUC), accuracy, F1 Score (F1), precision, and sensitivity as shown in Table 1.

**Table 1:** Accuracy values of different machine learning classifiers

	AUC	Accuracy	F1	Precision	Sensitivity
NB	91.7	83.3	83.3	83.3	83.3
LR	94.4	75.0	73.3	83.3	75.0
SGD	75.0	75.0	73.3	83.3	75.0

*AUC* area under ROC curve, *F1* F1 score, accuracy, precision, and sensitivity. *NB* naïve Bayes, *LR* logistic regression, *SGD* stochastic gradient descent

Values are presented as percentages

**Fig. 2:**
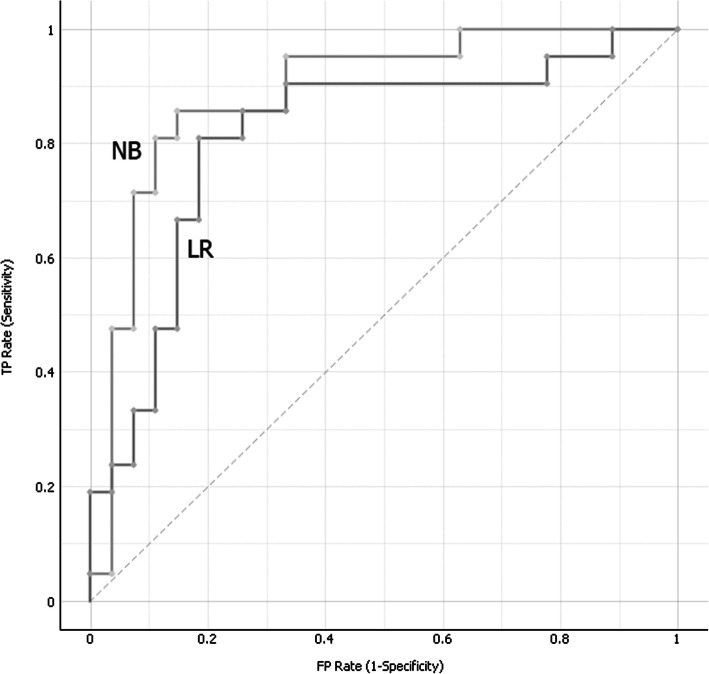
ROC (operating characteristic curve) curve for the two best ML (machine learning) classifiers, in which NB is naive Bayes and LR is logistic regression

Among the tested algorithms, naive Bayes (NB) obtained the highest accuracy (83.3%). While logistic regression (LR) and stochastic gradient descent (SGD) algorithms presented lower accuracy (75%). To determine the optimal cutoff value for both sensitivity and 1 minus specificity, we performed a plot of ROC curves for the three best classification methods, presented in Fig. 2.

Features selected through the NB classifier algorithm that obtained the highest diagnostic accuracy of 83.3% are reported in Table 2 with the gain ratio and Gini index, where we ranked the five best features for classification that consist of parameters derived from Wavelet’s transform and GLCM.

**Table 2:** Features selected by the NB classifier that obtained the highest diagnostic accuracy of 83.3%

Features	Gain ratio	Gini
Evhaar - 1	0.038	0.051
Evsym4 - 1	0.031	0.041
Evbior3.3 - 2	0.024	0.033
Kurtosis	0.019	0.026
Edsum4 - 2	0.015	0.020

Evhhar, Evsym, Evbior, and Edsum are the wavelet decomposition of the matrix *X* at level *N*, where *X* is the index after the wavelet name, and *N* is the index after the string “-”

### Clinical parameters

Table 3 shows the results of the clinical parameters to demonstrate knee conditions of all patients of the control and PRP groups in the periods before and 3 months after surgery.

**Table 3:** Clinical parameters of the control and PRP groups before and 3 months after surgery

Clinical parameters	Control group		PRP group	
	Before	After	Before	After
Circumference (cm)	40.6±3.4	41.2±4.2	42.6±4.2	43.4±4.5
Pain	5.8±1.6^f^	1.3±2.2^f^	4.9±2.4^g^	1.2±2.2^g^
Flexion (degrees - °)	137.3±11.3	140.6±8.9	134.6±13.6^e^	140.3±7.0^e^
IKDC index	49.5±16.5^a^	81.5±17.2^a^	46.1±11.9^b^	80.9±13.0^b^
Lysholm score	59.9±15.3^c^	88.2±21.6^c^	59.4±14.7^d^	91.1±10.7^d^

*IKDC* International Knee Documentation Committee

^a,b,c,d,e, f^, and ^g^ are significantly different with *p*<0.05

The first evaluated parameter was circumference, and there was no statistically significant difference neither before and after surgery nor among control and PRP groups. Regarding pain, there was a significant difference for each group, before and after surgery; however, no significant difference was found when comparing the control group and PRP group.

Analyzing flexion, in the control group, there was no significant difference before and after surgery. For the PRP group, there was a significant difference before and after surgery. However, when comparing the control group versus the PRP group, there was no significant difference. Since our statistical analysis was paired, the greater difference of flexion in the PRP group indicates a practical result of improved articulation.

Evaluating the index IKDC and Lysholm score, in the control group, both parameters presented statistically significant differences before and after surgery. The same difference was found for the PRP group before and after surgery, indicating a general improvement in the patients’ condition. However, when comparing the control versus the PRP groups, there were no significant difference, that is, the PRP did not improve the IKDC and Lysholm score results compared to control.

.

## Discussion

As observed in our findings, the texture analysis model combined with machine learning approaches presented very satisfactory results for differentiating the groups with and without PRP, especially with the naive Bayes (NB) classifier. Sample size was limited considering the retrospective nature of our study. Many patients were excluded from the study due to exclusion criteria and low adherence to clinical follow-up. Those results encourage us to establish that there is indeed a difference between patients that received PRP during ACL recovery surgery. And most importantly, the combined model of texture analysis (TA) and machine learning classifier (MLC) analysis are processed under 5 min, depending on the experience of the physician to identify the RoI. In our study, RoI positioning within the ACL was conducted by two experienced radiologists. They performed their analysis separately and their RoI area presented less than 2% of difference. This highlights the great potential to aid in medical diagnosis and reduce subjectivity in the assessment performed by the physicians.

To guarantee that there is a difference between the two groups (with and without PRP) represents a very important contribution to the discussion on whether PRP has any benefit in ACL surgery or not. For example, Figueroa et al. concludes that there is some evidence, regarding ACL reconstruction that PRP could be a synergic factor in acquiring maturity more quickly than graft with no PRP, however, the clinical implication of this remains unclear. Figueroa et al. included in the final analysis 11 papers (all published between 2005 and 2013).

According to Andriolo et al. the role of PRP use for ACL is controversial and did not provide superior clinical outcomes at short-term follow-up. Andriolo et al. included in the final analysis 32 papers (no time limitation). Di Matteo et al. concludes that data concerning the role of PRP are not conclusive to understand if it could provide faster recovery and better functional outcome during ACL repair/reconstruction. Despite some positive findings in terms of graft maturation and clinical outcome, further long-term studies are needed to identify whether the administration of PRP could truly play a beneficial role during ACL reconstruction. Di Matteo et al. included in the final analysis 21 papers (all published between 1996 and 2016).

In this context, with the development of our approach, we were able to demonstrate quantitatively that patients who used PRP in the ACL reconstruction surgery presented texture changes when compared to the group that did not receive PRP. This change was not visible to the eyes of radiologists but was identified through texture analysis in association with texture analysis and machine learning classifiers. Thus, we proved quantitatively that the PRP generated significant differences in texture. This can be used as a subsidy associated with clinical evaluations to assess improvement after the application of PRP.

Regarding the results of clinical parameters, for both control and PRP groups, there was an improvement in clinical parameters, except for circumference, when comparing knee conditions before and 3 months after reconstructive surgery. In particular, in the PRP group, flexion differences were significant after 3 months, while in the control group there was no significant difference, this may be an indication that PRP improved the degree of flexion in patients after surgery. However, when comparing the control group versus PRP, there was no significant difference between clinical parameters. These results are similar to those found in the literature [19–23].

Although clinically the patients showed no difference for the parameters when comparing control and PRP groups, the texture features extracted from MRI images showed a difference between the groups, mainly related to homogeneity and tissue healing within ACL. This may occur since clinical analyses and scores are subjective thus being more prone to errors due to human observation.

In the radiological evaluation, it was not possible to observe any difference between the control and PRP groups after 3 months of surgery. The images of the groups show similar patterns of ligament consolidation, with integral fillings and without injury. All patients in the control and PRP groups had ACL thickness with good definition and intact and fibrillary morphology, good tissue homogeneity, intact graft and without signs of injury, and with preservation of extension in both tibial (distal) and femoral (proximal) insertion with good preservation outlines.

## Conclusions

In conclusion, texture analysis (TA) associated with machine learning (ML) classifiers proved to be a feasible tool in the differentiation of patients after ACL reconstruction surgery with and without the use of PRP. Our results support that TA and ML could aid radiologists in classifying images, based on texture features that can be easily extracted from MRI images. We demonstrated quantitatively the occurrence of texture changes in patients that received PRP. Those results were confirmed with an accuracy of 83.3% and AUC of 94.4%. Thus, our findings suggest that PRP interferes with morphological parameters of the ACL reconstruction.

## Data Availability

The datasets used and/or analyzed during the current study are available from the corresponding author on reasonable request.

## Abbreviations

PRP: Platelet-rich plasma
ACL: Anterior cruciate ligament
MRI: Magnetic resonance imaging
ML: Machine learning
TA: Texture analysis
RoI: Regions of interest
LR: Logistic regression
NB: Naive Bayes
SGD: Stochastic gradient descent
IKDC: International Knee Documentation Committee
IRB: Institutional Review Board
RBC: Red blood cells
FFC: Frozen fresh plasma
PC: Platelet concentrate
PDH: Platelet-derived hormones
TSE: Turbo-spin-echo
GLCM: Gray-level co-occurrence matrix
GLRL: Gray-level run-length
ROC: Receiver operating characteristic curve
CA: Accuracy
F1: F score
AUC: Area under the ROC curve
